# Autophagosome maturation mediated by Rab7 contributes to neuroprotection of hypoxic preconditioning against global cerebral ischemia in rats

**DOI:** 10.1038/cddis.2017.330

**Published:** 2017-07-20

**Authors:** Lixuan Zhan, Siyuan Chen, Kongping Li, Donghai Liang, Xinyong Zhu, Liu Liu, Zhiwei Lu, Weiwen Sun, En Xu

**Affiliations:** 1 Institute of Neurosciences and Department of Neurology of The Second Affiliated Hospital of Guangzhou Medical University, Key Laboratory of Neurogenetics and Channelopathies of Guangdong Province and The Ministry of Education of China, Guangzhou 510260, China; 2 Department of Environmental Health, Rollins School of Public Health, Emory University, Atlanta 30322, Georgia

## Abstract

Autophagy disruption leads to neuronal damage in hypoxic–ischemic brain injury. Rab7, a member of the Rab GTPase superfamily, has a unique role in the regulation of autophagy. Hypoxic preconditioning (HPC) provides neuroprotection against transient global cerebral ischemia (tGCI). However, the underlying mechanisms remain poorly understood. Thus, the current study explored the potential molecular mechanism of the neuroprotective effect of HPC by investigating how Rab7 mediates autophagosome (AP) maturation after tGCI in adult rats. We found that HPC attenuated AP accumulation in the hippocampal CA1 region after tGCI via restoration of autophagic flux. We also confirmed that this HPC-induced neuroprotection was not caused by the increase in lysosomes or the improvement of lysosomal function after tGCI. Electron microscopic analysis then revealed an increase in autolysosomes in CA1 neurons of HPC rats. Moreover, the inhibition of autophagosome-lysosome fusion by chloroquine significantly aggravated neuronal death in CA1, indicating that AP maturation contributes to HPC-induced neuroprotection against neuronal injury after tGCI. Furthermore, the activation of Rab7 was found to be involved in the neuroprotective effect of AP maturation after HPC. At last, the knockdown of ultraviolet radiation resistance-associated gene (UVRAG) *in vivo* disrupted the interaction between Vps16 and Rab7, attenuated the activation of Rab7, interrupted autophagic flux, and ultimately abrogated the HPC-induced neuroprotection against tGCI. Our results indicated that AP maturation was enhanced by the activation of Rab7 via UVRAG-Vps16 interaction, which further demonstrated the potential neuroprotective role of Rab7 in HPC against tGCI-induced neuronal injury in adult rats.

Transient global cerebral ischemia (tGCI) can be caused by drowning, cardiac arrest or cardiopulmonary bypass surgery, thus leading to delayed neuronal death in the hippocampal CA1 subregion. Ischemic preconditioning (IPC) confers neuroprotection of CA1 pyramidal neurons against a subsequent severe ischemic injury.^1^ We previously reported that hypoxia preconditioning (HPC) with 8% oxygen for 30–120 min applied 1–4 days before ischemia reduced cell death in CA1 after tGCI.^2^ However, the molecular mechanisms underlying ischemic tolerance induced by HPC remain incompletely understood.

Autophagy is a process that degrades intracellular organelles and long-lived cytosolic proteins to maintain cell homeostasis. It includes four stages: initiation, elongation, maturation and degradation.^3, 4^ Autophagy is initiated by forming a double-membraned compartment known as an autophagosome (AP). APs can fuse with lysosomes to produce autolysosomes (ALs), which allows APs to obtain hydrolytic enzymes indispensable for the subsequent autophagic degradation.^5^ This process is known as AP maturation. Defects in AP maturation or AP clearance can cause accumulation of APs within a cell, which can bring about serious disorders.^6, 7, 8^ Particularly, autophagic dysfunction in neurons is associated with various neurodegenerative diseases, including Alzheimer’s disease, Parkinson’s disease and Huntinton’s disease.^9, 10^ However, it is still unclear whether AP maturation participated in HPC-induced neuroprotection against tGCI.

AP maturation is modulated by multiple factors, such as E1 ubiquitin activating enzyme, Cathepsin D and small GTP-binding protein Rab7 (Ref. 6, 11), among which only Rab7 is crucial for complete autophagic flux.^12^ Rab7, a small 208-amino acid protein in the family of GTPases, is predominantly detected in late endosomes.^13^ It can promote lysosome biogenesis and maintain lysosome’s function.^14^ Rab7 has a key role in catalyzing the fusion of vacuoles with lysosomes,^15^ and in AP maturation. Mutations in Rab7, as well as its abnormal expression and activity, might be associated with neurodegenerative diseases, lipid storage disorders and heart diseases.^16, 17, 18^ In addition, the downregulation of Rab7 induced by myocardial ischemia would lead to disturbance of AP maturation.^19^ However, little is known about the roles and the molecular mechanisms of Rab7 in AP maturation after cerebral ischemia.

The activity of Rab7 depends on its active (GTP-bound) and inactive (GDP-bound) states.^20^ The GDP/GTP cycle of Rab7 is regulated by two interrelated proteins, the guanine nucleotide exchange factors (GEFs) and GTPase-activating proteins.^21, 22^ GEFs mediate the activation of Rab7 by switching from GDP to GTP form. Collins *et al.*^23^ reported that the homotypic fusion and vacuole protein sorting (HOPS) complex interacts with Ypt-Rab GTPase and activates Ypt7p (the yeast Rab7 orthologue) during membrane fusion.The HOPS complex consists of six subunits, including the Class B vacuolar protein sorting (Vps) complex and class C Vps (C-Vps) complex. The latter contains Vps11, Vps16, Vps18 and Vps33. C-Vps complex acts as a GEF of Rab7 and promotes the activation of Rab7.^24^ However, the function of C-Vps complex on autophagy remains less established.

Existing studies demonstrate that the C-Vps complex can be positively regulated by the ultraviolet radiation resistance-associated gene (UVRAG).^25^ The UVRAG consists of four major regions: the proline-rich domain, the calcium-dependent lipid-binding C2 domain, the coiled-coil domain and a C-terminal domain. C2 domain is required for UVRAG-mediated membrane-lipid association,^25^ and the deletion of this domain disrupts UVRAG’s interaction with the C-Vps complex, thereby impairing the ability of UVRAG in promoting AP fusion with lysosomes.^25^ Meanwhile, UVRAG-C-Vps interaction potentiates GDP/GTP exchange of Rab7, which in turn promotes endosome maturation.^25, 26^ However, whether the UVRAG-C-Vps interaction can activate Rab7 during AP maturation remains to be clarified.

In this study, we sought to investigate the impact of AP maturation deficiency on neuronal damage after tGCI and to examine a potential link between the Rab7 activation and AP maturation in HPC-induced ischemic tolerance. Furthermore, we tested a hypothesis that UVRAG-C-Vps interaction enhances the ability of the C-Vps complex to activate Rab7, which ultimately promotes AP maturation to protect neurons from tGCI-induced damage after HPC.

## Results

### HPC decreases the accumulation of APs in CA1 after tGCI

**Figure 1:**
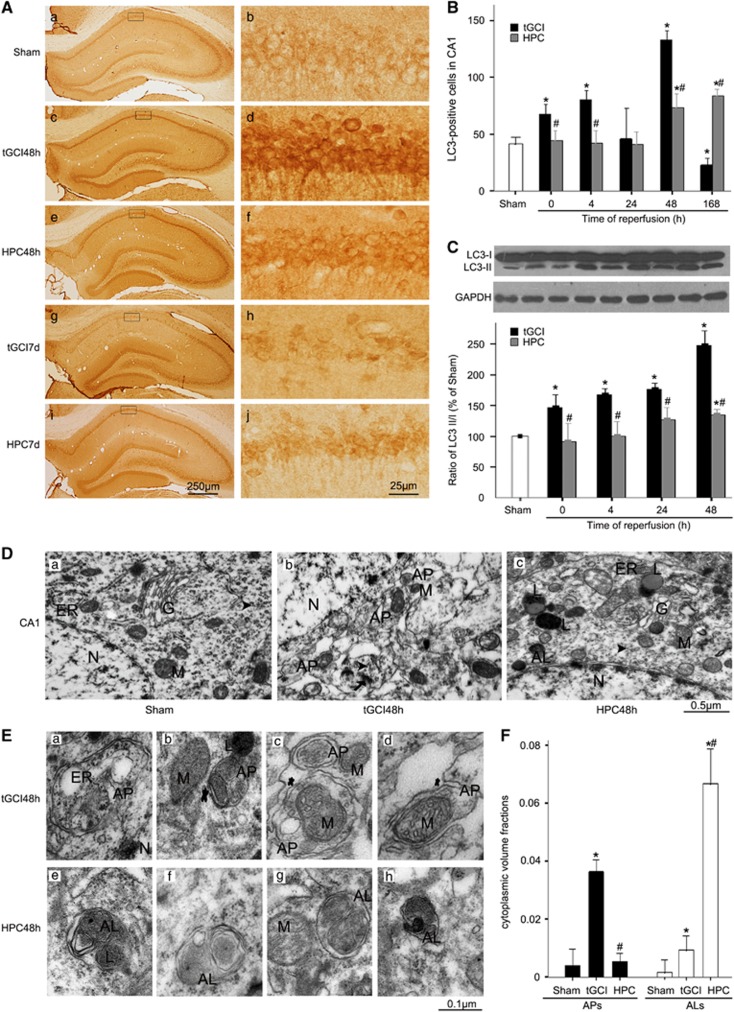
Hypoxic preconditioning decreases AP accumulation in hippocampal CA1 region after tGCI. (**A**) Immunohistochemistry for LC3 in the hippocampus after tGCI with or without HPC. Representative images show Sham-operated group (a and b), 48 h after reperfusion of tGCI group (c and d), 48 h after reperfusion of HPC group (e and f), 7 days after reperfusion of tGCI group (g and h), and 7 days after reperfusion of HPC group (i and j), respectively. Scale bar: a, c, e, g, i: 250 *μ*m; b, d, f, h, j: 25 *μ*m. (**B**) Quantitative analysis of immunoreactive cell counting of LC3 in CA1. Data are shown as mean±S.D. *P<0.05 *versus* Sham-operated group and ^#^*P*<0.05 *versus* tGCI group at the same time point (*n*=6 in each group). (**C**) Western blot analysis of LC3 in CA1 of ischemic and hypoxic preconditioned rats. The histogram presents the quantitative analyses of the ratio of LC3-II/LC3-I. Data are expressed as percentage of value of Sham-operated animals. *P<0.05 *versus* Sham-operated animals and ^#^*P*<0.05 *versus* tGCI group at the same time point (*n*=5 in each group). (**D**) TEM micrographs of CA1 neurons in ischemic and hypoxic preconditioned rats. CA1 neurons from Sham-operated rats (a) contained normal polyribosomes (arrowheads), nucleus (N), mitochondria (M), endoplasmic reticulum (ER) and Golgi apparatus (G). CA1 neurons from brains subjected to 10 min of ischemia followed by 48 h reperfusion (b) displayed dissociation of polyribosomes, fragmentation of the neuronal Golgi apparatus and marked accumulation of intracellular vesicles and protein aggregates (arrows), as well as a dramatic increase in APs. Most abnormal morphological changes such as protein aggregates and dissociation of polyribosomes were absent in CA1 neurons from HPC group at 48 h after reperfusion (c), whereas moderate dilated endoplasmic reticulum and swollen mitochondria, and an obviously increase in ALs were observed. Scale bar: 0.5 *μ*m. (**E**) TEM micrographs of autophagic ultrastructure in CA1 neurons from ischemic and hypoxic preconditioned rats at 48 h after reperfusion. The APs can be identified by its contents (morphologically intact cytoplasm, including ribosomes and ER), and a limiting membrane that is partially visible as two bilayers separated by a narrow elecron-lucent cleft, that is, as a double membrane (asterisk). a: one double-membraned cistern, containing ribosomes and dilated ER; b: an AP containing two mitochondrion and membranous structures; c: an AP containing vesicles and an AP containing dilated or damaged mitochondrion; d: an AP containing dilated or damaged mitochondrion. ALs typically have only one limiting membrane; frequently they contain electon dense cytoplasmic materials and/or organelles at various stages of degradation. e: an AL with partially digested organelles and a lysosome. f–h: ALs partially digested mitochondrion and cytoplasmic materials. Scale bar: 0.1 *μ*m. (**F**) The cytoplasmic volume fractions of APs and ALs in CA1 neurons from ischemic and hypoxic preconditioned rats. Data expressed as percentage±S.D. show the cytoplasmic volume fractions of APs and ALs. *P<0.05 *versus* Sham-operated group and ^#^*P*<0.05 *versus* tGCI group at the same time point (*n*=5 in each group)

To examine the activity of autophagy, immunohistochemistry and western blot of microtubule-associated protein 1 light chain 3 (LC3) in CA1 were performed. The number of LC3-positive cells was significantly higher at 0–4 h, peaked at 48 h, and decreased by day 7 after tGCI as compared with Sham. Contrary to tGCI rats, the change of LC3 immunoreactivity was reversed at each time point described above in HPC groups (Figure 1A and B). We further confirmed that the LC3-II/LC3-I ratio in CA1 increased in time-dependent manner after tGCI, and this increase was reversed by HPC (Figure 1C).

Next, we examined the brain sections by transmission electron microscopy (TEM). Neurons from CA1 in Sham-operated group displayed normal nucleus, mitochondria, endoplasmic reticulum and Golgi apparatus (Figure 1D, a), whereas neurons in tGCI group demonstrated a remarkable increase in APs, protein aggregate, dilated endoplasmic reticulum and swollen mitochondria after 48 h of reperfusion (Figure 1D, b and Figure 1E, a–d). Seven days after tGCI, neurons exhibited morphologic characteristics of necrotic cell death such as amorphous organelles and cytoplasm disorganization (data not shown). In contrast to tGCI groups, although a dilated endoplasmic reticulum and swollen mitochondria were observed, there seemed to be significantly more ALs in HPC groups (Figure 1D, c and Figure 1E, e–h). The cytoplasmic volume fractions of recognizable APs and ALs within the defined CA1 were counted at 48 h of reperfusion. Relative to Sham-operative rats, the cytoplasmic volume fractions of APs largely increased after tGCI, but this increase was reversed with HPC. Interestingly, the cytoplasmic volume fractions of ALs substantially increased in HPC group compared with tGCI group (Figure 1F).

### HPC decreases the accumulation of APs via restoration of autophagic flux after tGCI

To investigate the potential mechanism of autophagy after tGCI with HPC, we examined the expression of autophagy-related protein 5 (Atg5) (Supplementary Fig. I in the Supplementary Material). Atg5-positive cells from Sham animals had round nuclei and spindle cell bodies with elongated axons, showing a typical neuron-like morphology. Alternatively, Atg5 staining appeared in cells with elongated and irregular nuclei at 168 h after tGCI. Quantitative analysis showed no significant difference in the number of Atg5-immunopositive cells in CA1 from ischemic or HPC brains up to 168 h of reperfusion. Similar results were obtained in western blot. These data indicates that decreased AP accumulation induced by HPC was not due to the suppression of autophagy initiation.

**Figure 2:**
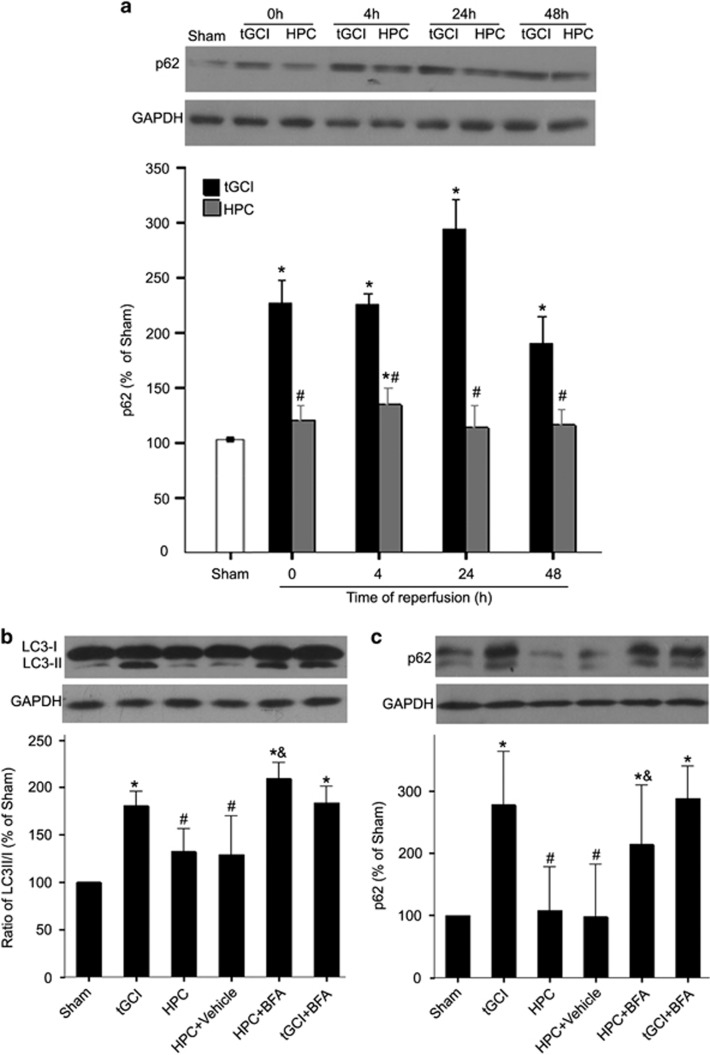
Hypoxic preconditioning restores autophagic flux after tGCI. (**a**) Western blot analysis of SQSTM1/p62 in CA1 of ischemic and hypoxic preconditioned rats. The histogram presents the quantitative analyses of SQSTM1/p62 levels. Data are expressed as percentage of value of Sham-operated animals. Each bar represents the mean±S.D.*P<0.05 *versus* Sham-operated animals and ^#^*P*<0.05 *versus* tGCI group at the same time point (*n*=7 in each group). (**b**) Effects of BFA on the expression of LC3 in ischemic and hypoxic preconditioned rats analyzed by western blot. Rats were treated with 2.5 *μ*g of BFA or Vehicle intracerebroventricularly at 30 min before tGCI or hypoxia. The same amount of protein lysates from CA1 was subjected to immunoblot analysis using anti-LC3 antibody at 24 h after reperfusion. The histogram presents the quantitative analyses of the ratio of LC3-II/LC3-I. (**c**) Effects of BFA on the expression of SQSTM1/p62 in ischemic and hypoxic preconditioned rats analyzed by western blot. The histogram presents the quantitative analyses of SQSTM1/p62 levels. Data are expressed as percentage of value of Sham-operated animals. The values are expressed as mean±S.D. *P<0.05 *versus* Sham-operated animals, ^#^*P*<0.05 *versus* tGCI group and ^&^*P*<0.05 *versus* HPC+Vehicle group (*n*=7 in each group). p62, SQSTM1/p62

Then, we investigated whether the restoration of autophagic flux contributed to the decrease of AP accumulation by HPC. SQSTM1/p62 increased markedly through 48 h of reperfusion after tGCI with a time to peak of 24 h. In contrast, HPC eliminated the effects in the expression of SQSTM1/p62 induced by tGCI (Figure 2a). Moreover, both the LC3-II/LC3-I ratio and SQSTM1/p62 in CA1 remarkably increased at 24 h after reperfusion of HPC group with bafilomycin-A1 (BFA) pretreatment. However, these increases induced by BFA pretreatment were relatively less substantial in tGCI group (Figures 2b and c). These results indicate that HPC restored autophagic flux after tGCI.

### HPC induces neuroprotection against tGCI via promoting AP maturation

**Figure 3:**
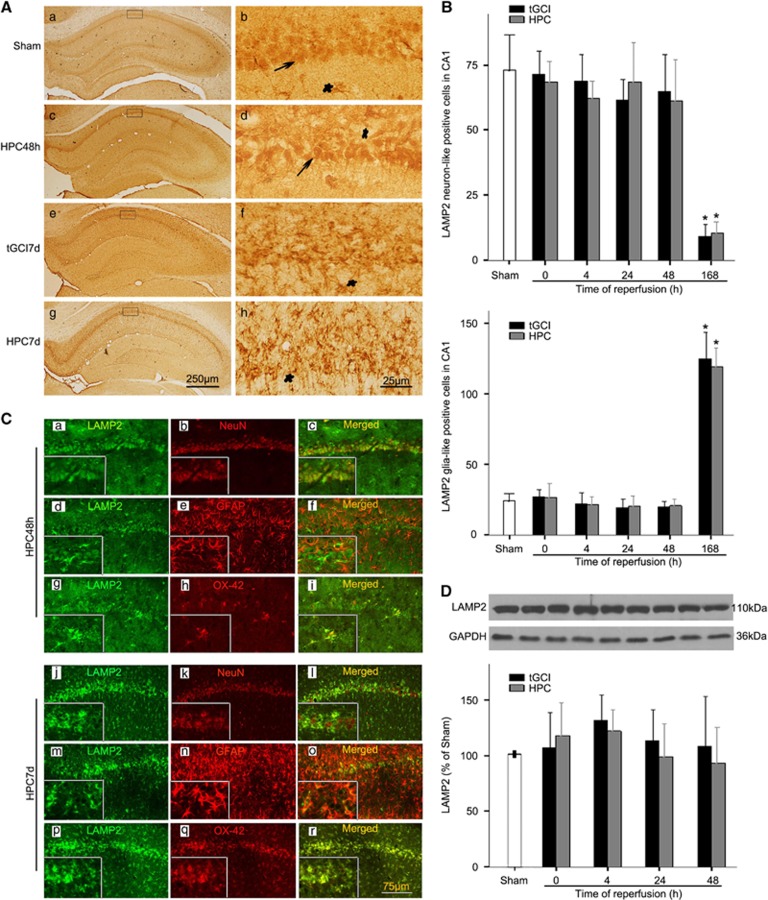
The effect of hypoxic preconditioning on the protein expression of LAMP2 in CA1 after tGCI. (**A**) Immunohistochemistry for LAMP2 in the hippocampus after tGCI with or without HPC. Representative images show Sham-operated group (a and b), 48 h after reperfusion of HPC group (c and d), 7 days after reperfusion of tGCI group (e and f), and 7 days after reperfusion of HPC group (g and h), respectively. LAMP2-positive neuron-like cells had rounded nuclei, and spindle cell body with elongated axon (arrow) and LAMP2-positive glia-like cells appeared mostly in cells with elaborate array of processes and irregular nuclei (asterisk). Scale bar: a, c, e, g: 250 *μ*m; b, d, f, h: 25 *μ*m. (**B**) Quantitative analysis of immunoreactive cell counting of LAMP2-positive neuron-like cells and LAMP2-positive glia-like cells in the CA1 subregion. Data are shown as mean±S.D. *P<0.05 *versus* Sham-operated animals (*n*=6 in each group). (**C**) Representative photomicrographs show fluorescent double staining of LAMP2 and NeuN (red), LAMP2 (green) and GFAP (red) and LAMP2 (green) and OX-42 (red) in the rat brains at 48 h and 7 days after tGCI with hypoxia, respectively. The overlapped images show that the vast majority of LAMP2 was colocalized with NeuN (c), and only minority of LAMP2 overlapped with OX-42 at 48 h after reperfusion (i). However, LAMP2 and OX-42 were almost overlapped at 7 days after reperfusion. Scale bar: 75 *μ*m. (**D**) Western blot analysis of LAMP2 in CA1 of ischemic and hypoxic preconditioned rats. The histogram presents the quantitative analyses of LAMP2 levels. Data are expressed as percentage of value of Sham-operated animals. Each bar represents the mean±S.D. (*n*=5 in each group)

The accumulation of autophagic vacuoles could result from three sources, a selective impairment in autophagosome-lysosome fusion, the dysfunction of lysosomal proteolysis, or a decrease in the number of lysosomes. Accordingly, we measured the levels of lysosomal-associated membrane protein-2 (LAMP2) and Cathepsin D, respectively. Most LAMP2-positive cells from the Sham-operated rats had round nuclei with a granular appearance (Figure 3A), and double-fluorescent immunohistochemistry revealed that these cells were neuronal nuclei (NeuN)-positive. Also, only a minority of LAMP2-positive cells were complement receptor type 3 (OX-42)-positive, which displayed an irregular appearance with polymorphic somata and processes (data not shown). Our results showed no significant differences in the number and distribution of LAMP2-positive cells in CA1 between ischemic and HPC brains until 48 h (Figure 3B). Similar results were observed in western blot (Figure 3D). Alternatively, at 168 h after ischemia, LAMP2 staining mainly existed in cells which exhibited the typical features of activated microglia (Figure 3A, e–h). The results from double-fluorescent immunohistochemistry supported the predominant localization of LAMP2 in microglia (Figure 3C, p–r).

**Figure 4:**
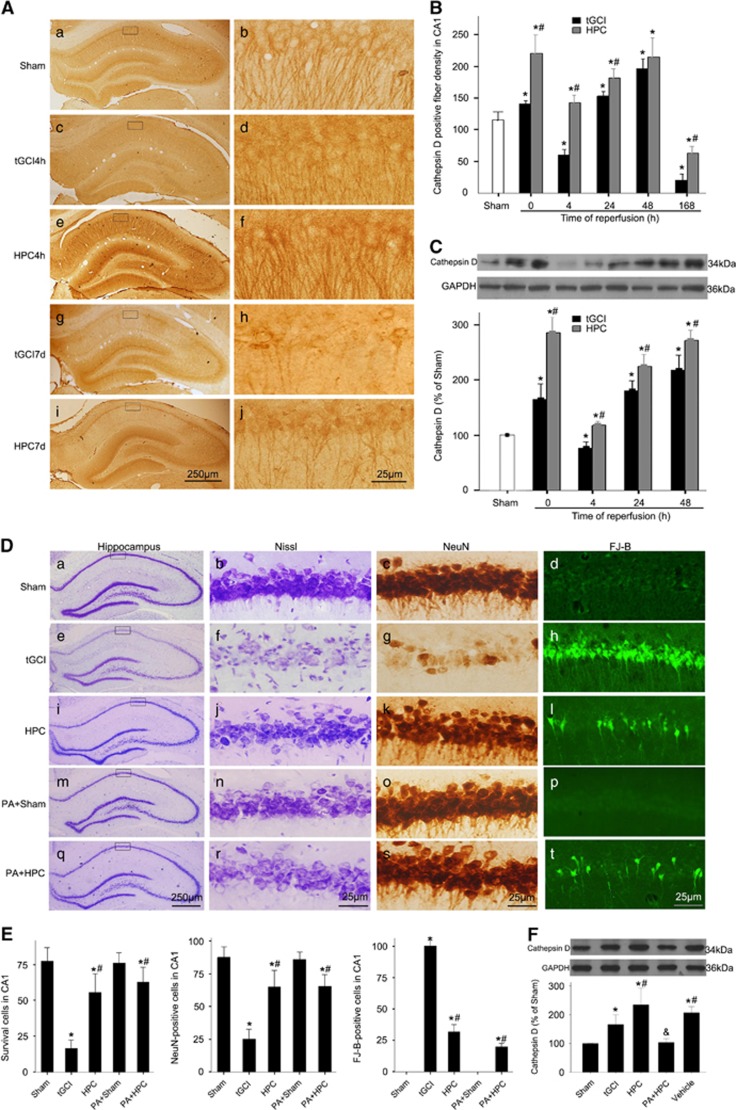
Hypoxic preconditioning improves lysosomal function after tGCI. (**A**) Immunohistochemistry for Cathepsin D in the hippocampus after tGCI with or without HPC. Representative images show Sham-operated group (a and b), 4 h after reperfusion of tGCI group (c and d), 4 h after reperfusion of HPC group (e and f), 7 days after reperfusion of tGCI group (g and h), and 7 days after reperfusion of HPC group (i and j), respectively. Scale bar: a, c, e, g, i: 250 *μ*m; b, d, f, h, j: 25 *μ*m. (**B**) Quantitative analysis of Cathepsin D-positive fiber density in CA1. Data are shown as mean±S.D. *P<0.05 *versus* Sham-operated group and ^#^*P*<0.05 *versus* tGCI group at the same time point (*n*=6 in each group). (**C**) Western blot analysis of Cathepsin D in CA1 of ischemic and hypoxic preconditioned rats. The histogram presents the quantitative analyses of Cathepsin D levels. Data are expressed as percentage of value of Sham-operated animals. Each bar represents the mean±S.D. *P<0.05 *versus* Sham-operated animals and ^#^*P*<0.05 *versus* tGCI group at the same time point (*n*=5 in each group). (**D**) Representative microphotographs of cresyl violet staining, immunostaining of NeuN and FJ-B staining in the hippocampus at 7 days after HPC with or without PA treatment. Sham group (a–d); tGCI group (e–h); HPC group (i–l); PA+Sham group (m–p), pretreatment with PA without ischemia or hypoxia; PA+HPC group, pretreatment with PA at 2 h before HPC (q–t). Scale bar: a, e, i, m, q: 250 *μ*m, b–d, f–h, j–l, n–p, r–t: 25 *μ*m. (**E**) Quantitative analyses of survival neurons, NeuN-positive cells and FJ-B-positive cells in CA1. Each bar represents the mean±S.D. *P<0.05 *versus* Sham-operated animals and ^#^*P*<0.05 *versus* tGCI group (*n*=6 in each group). (**F**) Effects of PA on Cathepsin D in HPC rats. Rats were treated with PA intraperitoneally at 2 h before HPC. The same amount of protein lysates from CA1 were subjected to immunoblot analysis using anti-Cathepsin D antibody at 0 h of reperfusion. The values are expressed as mean±S.D. *P<0.05 *versus* Sham-operated animals and ^#^*P*<0.05 *versus* tGCI group and ^&^*P*<0.05 *versus* Vehicle group (*n*=5 in each group)

Unlike LAMP2, the levels of Cathepsin D sharply increased beside a transient decrease at 4 h after tGCI. However, compared with tGCI groups, a persistent increase of Cathepsin D was detected in HPC groups (Figures 4A–C). These data implied that the decreased Cathepsin D in the early stage after ischemia may have led to lysosomal malfunction and thus potentially contributed to the neuronal damage. Accordingly, we evaluated the effects of Pepstatin A (PA), the Cathepsin D inhibitor, on the neuronal death after HPC. Compared with the neuronal morphology in Sham-operated group (Figure 4D, a–d), significant neuronal damage in CA1 was observed in tGCI group (Figure 4D, e–h, and Figure 4E). In HPC group, survival cells and NeuN-positive cells sharply increased, whereas Fluoro-Jade B (FJ-B)-positive cells markedly decreased compared with tGCI rats (Figure 4D, i–l and Figure 4E). Interestingly, after confirming that PA had no neurotoxic effects on the cells of hippocampus in Sham-operated rats (Figure 4D, m–p, and Figure 4E), we further demonstrated that pretreatment with PA did not block the neuroprotection of HPC (Figure 4D, q–t, and Figures 4E and F). Taken together, our results suggest that the neuroprotection induced by HPC may not have resulted from the increase in lysosome number or the improvement of lysosomal function.

**Figure 5:**
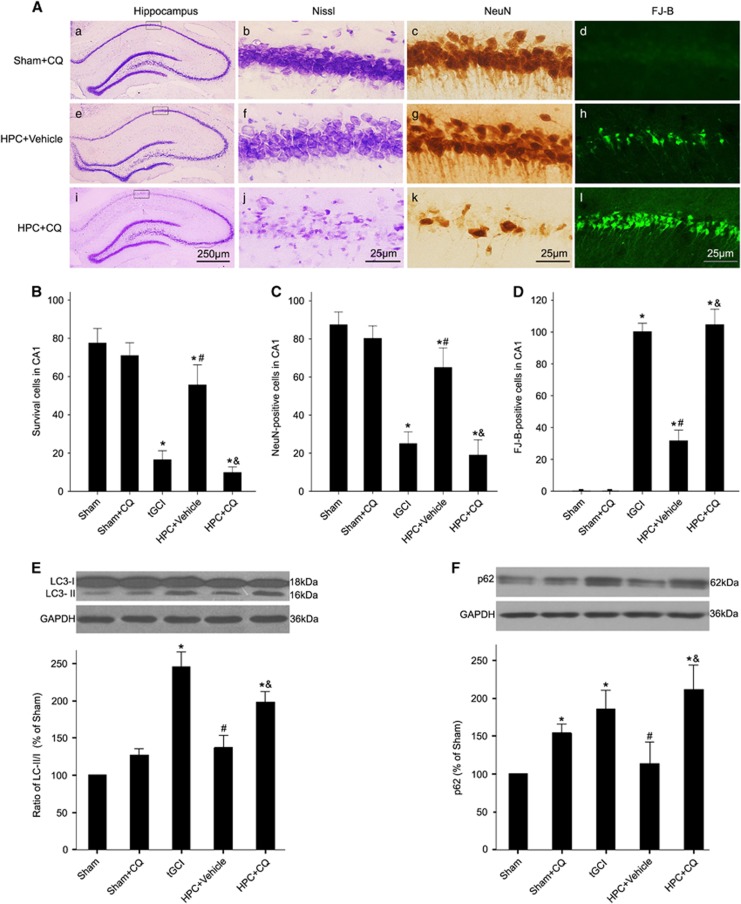
Impaired AP maturation by pretreatment with chloroquine causes neuronal damage in CA1 of hypoxic preconditioned rats. (**A**) Representative microphotographs of cresyl violet staining, immunostaining of NeuN, and FJ-B staining in the hippocampus at 7 days after HPC with or without CQ treatment. Sham+CQ group (a–d), pretreatment with CQ without ischemia or hypoxia; HPC+Vehicle group (e–h), pretreatment with normal saline at 2 h before HPC; HPC+CQ group (i–l), pretreatment with CQ at 2 h before HPC. Scale bar: a, e, i: 250 *μ*m, b–d, f–h, j–l: 25 *μ*m. (**B**–**D**) Quantitative analyses of survival neurons, NeuN-positive cells and FJ-B-positive cells in CA1. Each bar represents the mean±S.D. *P<0.05 *versus* Sham-operated animals, ^#^*P*<0.05 *versus* tGCI group and ^&^*P*<0.05 *versus* HPC+Vehicle group (*n*=6 in each group). (**E**) Effects of CQ on LC3 in hypoxic preconditioned rats. Rats were treated with 60 mg/kg of CQ or vehicle intracerebroventricularly at 2 h before hypoxia. The same amount of protein lysates from CA1 was subjected to immunoblot analysis using anti-LC3 antibody at 48 h after reperfusion. The histogram presents the quantitative analyses of the ratio of LC3-II/LC3-I. The values are expressed as mean±S.D. *P<0.05 *versus* Sham-operated animals, ^#^*P*<0.05 *versus* tGCI group and ^&^*P*<0.05 *versus* HPC+Vehicle group (*n*=5 in each group). (**F**) Western blot analysis of SQSTM1/p62 in CA1 of hypoxic preconditioned rats after pretreatment with CQ. The histogram presents the quantitative analyses of SQSTM1/p62 levels. Data are expressed as percentage of value of Sham-operated animals. The values are expressed as mean±S.D. *P<0.05 *versus* Sham-operated animals, ^#^*P*<0.05 *versus* tGCI group and ^&^*P*<0.05 *versus* HPC+Vehicle group (*n*=6 in each group). CQ: chloroquine

Similarly, to further ascertain whether the promotion of autophagosome-lysosome fusion have contributed to HPC-induced neuroprotection against tGCI, rats were pretreated with chloroquine (CQ) (Figure 5), which inhibits autophagosome-lysosome fusion.^27^ The pretreatment with CQ before HPC completely eliminated the neuroprotective effect of HPC, and increased LC3-II and SQSTM1/p62 in CA1 after tGCI. These results support that HPC restores autophagic flux owing to the promotion of autophagosome-lysosome fusion, which in turn contributes to neuroprotection against neuronal injury after tGCI.

### HPC induces AP maturation via activating Rab7 after tGCI

**Figure 6:**
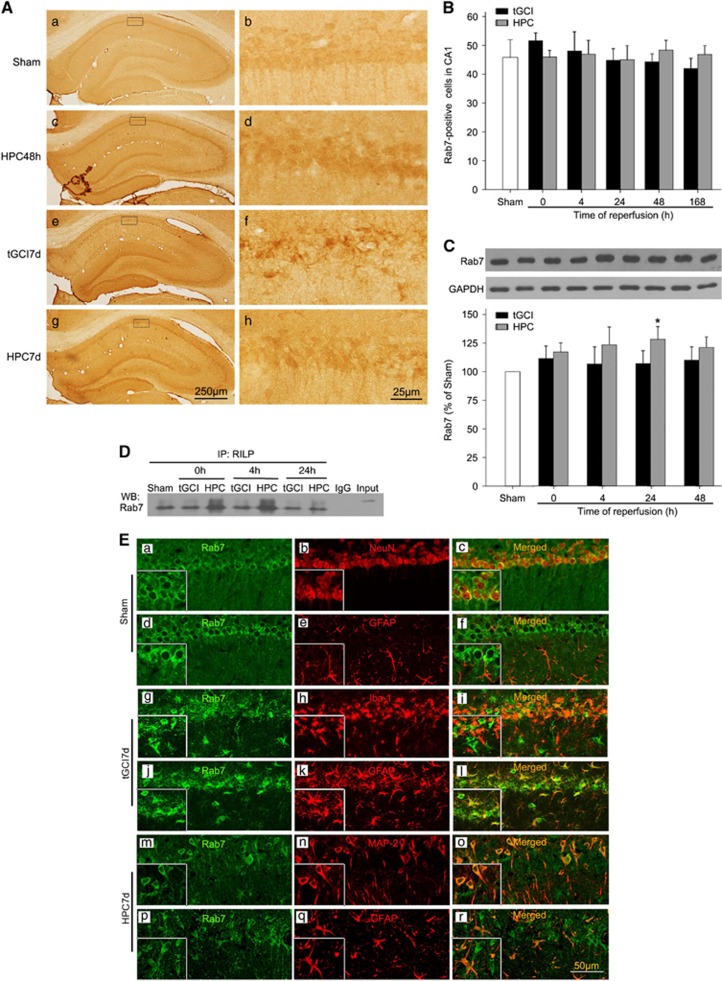
Hypoxic preconditioning increases the activation of Rab7 in hippocampal CA1 region after tGCI. (**A**) Immunohistochemistry for Rab7 in the hippocampus after tGCI with or without HPC. Representative images show Sham-operated group (a and b), 48 h after reperfusion of HPC group (c and d), 7 days after reperfusion of tGCI group (e and f), and 7 days after reperfusion of HPC group (g and h), respectively. Scale bar: a, c, e, g: 250 *μ*m; b, d, f, h : 25 *μ*m. (**B**) Quantitative analysis of immunoreactive cell counting of Rab7 in CA1. Data are shown as mean±S.D. (*n*=6 in each group). (**C**) Western blot analysis of Rab7 in CA1 of ischemic and hypoxic preconditioned rats. The histogram presents the quantitative analyses of Rab7 levels. Data are expressed as percentage of value of Sham-operated animals. Each bar represents the mean±S.D.*P<0.05 *versus* Sham-operated animals (*n*=6 in each group). (**D**) Immunoprecipitation blots showing the Rab7-RILP complex in CA1 of ischemic and hypoxic preconditioned rats. RILP was immunoprecipitated (IP) using anti-RILP antibody. Rab7 was detected by western blot (WB). The experiments were repeated twice (*n*=3 in each group). (**E**) Representative photomicrographs show fluorescent double staining of Rab7 (green) and NeuN (red), Rab7 (green) and GFAP (red), Rab7 (green) and Iba-I (red) and Rab7 (green) and MAP-2 (green) in the rat brains, respectively. The overlapped images show that Rab7 surrounded NeuN in Sham (c). However, Rab7 located mainly in Iba-1 and GFAP-positive cells in CA1 at 168 h after tGCI (i, l). Alternative, Rab7 located mainly in MAP-2 and GFAP-positive cells in CA1 at 168 h after HPC (i, l) Scale bar: 50 *μ*m

To explore the molecular mechanisms underlying AP maturation in HPC rats, we measured the expression of Rab7 in CA1. Rab7-positive cells from Sham-operated rats had large spindle-shaped soma with single apical axon (Figure 6 A, a–b). Double-fluorescent immunohistochemistry revealed that they surrounded NeuN-positive cells (Figure 6E, a–c). No colocalization of Rab7 with glial fibrillary acidic protein (GFAP) was found (Figure 6E, d–f). Notably, at 168 h of reperfusion after tGCI, Rab7 located mainly in cells with elongated and irregular nuclei (Figure 6A, e–f), and double-fluorescent immunohistochemistry revealed that they were positive for ionized calcium binding adaptor molecule 1 (Iba-1) (Figure 6E, g–i) and GFAP (Figure 6E, j–l). Alternatively, most Rab7-positive cells in HPC rats had large spindle-shaped soma with single apical axon (Figure 6A, g–h). Double-fluorescent immunohistochemistry revealed that they were microtubule-associated protein-2 (MAP-2)-positive (Figure 6E, m–o). There were no significant differences in the number of Rab7-positive cells between ischemic and HPC brains up to 168 h of reperfusion (Figures 6A and B). Similar results were observed in western blot (Figure 6C). These results indicate that the alteration of Rab7 protein may not have been involved in the effect of AP maturation after HPC. We further examined the activity of Rab7 using a Rab7 effector’s protein co-immunoprecipitation assay.^28^ The results showed that Rab7-interacting lysosomal protein (RILP) interacting with Rab7 in HPC rats was substantially higher than that in tGCI rats, especially at 0–4 h after reperfusion (Figure 6D). These data indicates that the activation of Rab7 after HPC may have facilitated AP maturation.

### HPC activates Rab7 via mediating UVRAG-Vps16 interaction after tGCI

**Figure 7:**
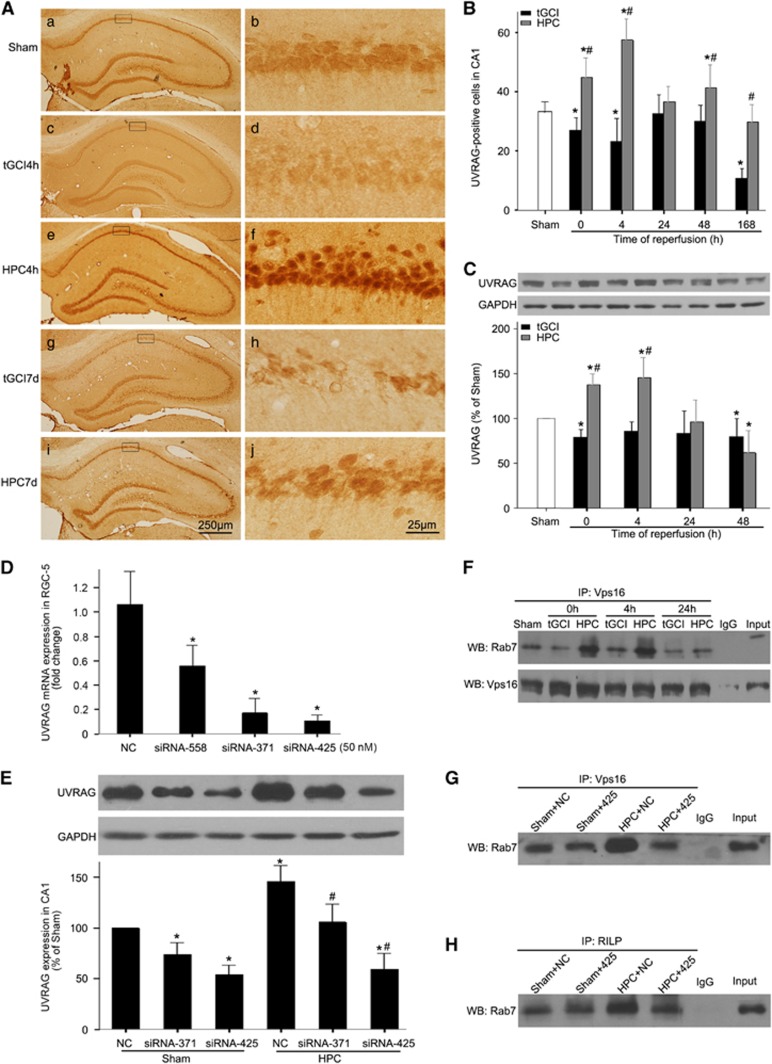
Hypoxic preconditioning increases the expression of UVRAG and enhances the ability of the C-Vps complex to activate Rab7 in hippocampal CA1 region after tGCI. (**A**) Immunohistochemistry for UVRAG in the hippocampus after tGCI with or without HPC. Representative images show Sham-operated group (a and b), 4 h after reperfusion of tGCI group (c and d), 4 h after reperfusion of HPC group (e and f), 7 days after reperfusion of tGCI group (g and h), and 7 days after reperfusion of HPC group (i and j), respectively. Scale bar: a, c, e, g, i: 250 *μ*m; b, d, f, h, j: 25 *μ*m. (**B**) Quantitative analysis of immunoreactive cell counting of UVRAG in CA1. Data are shown as mean±S.D. *P<0.05 *versus* Sham-operated group and ^#^*P*<0.05 *versus* tGCI group at the same time point (*n*=6 in each group). (**C**) Western blot analysis of UVRAG in CA1 of ischemic and hypoxic preconditioned rats. The histogram presents the quantitative analyses of UVRAG levels. Data are expressed as percentage of value of Sham-operated animals. Each bar represents the mean±S.D. *P<0.05 *versus* Sham-operated animals and ^#^*P*<0.05 *versus* tGCI group at the same time point (*n*=6 in each group). (**D**) RT-qPCR showed that UVRAG mRNA expression in RGC-5 was significantly downregulated after transfection with siRNAs, and siRNA-371 and siRNA-425 at 50 nM presented the higher efficacy. *P<0.05 *versus* negative control (NC, *n*=3 in each group). (**E**) Representative picture from western blot showed that protein expressions of UVRAG in CA1 of Sham-operated and hypoxic preconditioned rats were remarkably decreased after transfection with siRNAs into the dorsal CA1 pyramidal layer of rat brains. Data are expressed as percentage of value of Sham+NC animals. Each bar represents the mean±S.D. *P<0.05 *versus* Sham+NC animals and ^#^*P*<0.05 *versus* HPC+NC group (*n*=6 in each group). (**F**) Immunoprecipitation blots showing the interaction of Vps16 with Rab7 in CA1 of ischemic and hypoxic preconditioned rats. Vps16 was immunoprecipitated using anti-Vps16 antibody. Rab7 was detected by WB. (**G**–**H**) The effects of UVRAG knockdown on the interaction of Vps16 with Rab7 and the activation of Rab7. The same amount of protein lysates from CA1 were obtained at 4 h after reperfusion in HPC or 24 h after injection of vectors in Sham-operated group. RILP was immunoprecipitated using anti-RILP antibody. Rab7 was detected by WB. The experiments were repeated twice (*n*=3 in each group)

We then hypothesized that UVRAG and its modulation by Vps16 were required for the activation of Rab7 after HPC. Our results showed that UVRAG in tGCI groups was remarkably downregulated after reperfusion compared with the Sham-operated group. However, in HPC groups, it increased immediately after reperfusion, and continued to increase up to 4 h and then returned to the Sham level at 24 h post-ischemia (Figure 7A–C). To investigate the involvement of UVRAG in the activation of Rab7, we performed small-interfering RNA (siRNA)-mediated knockdown of UVRAG. The silencing efficacy of siRNAs *in vitro* is shown in Figure 7D. UVRAG-rat-371 and UVRAG-rat-425 had higher silencing efficacy in *in vitro* study with retinal ganglion cells (RGC-5), which was determined by reverse transcription quantitative real-time polymerase chain reaction (RT-qPCR). Then, UVRAG-rat-371 and UVRAG-rat-425 were selected to combine with HVJ-Envelope (HVJ-E) to transfer into the dorsal CA1 pyramidal layer of rats. The expression of UVRAG in CA1 detected by western blot significantly decreased after the administration of UVRAG siRNAs either in Sham-operated or in HPC rats (Figure 7E). Moreover, the expression of UVRAG with the vector of UVRAG-rat-425 was lower than that of UVRAG-rat-371.

Next, interaction of Vps16 with Rab7, assessed by co-immunoprecipitation, was enhanced in CA1 after HPC (Figure 7F). The strength of this interaction was weakened in UVRAG knockdown rats (Figure 7G). Furthermore, the activation of Rab7 was largely reduced by UVRAG knockdown either in HPC or Sham-operated rats (Figure 7H).

**Figure 8:**
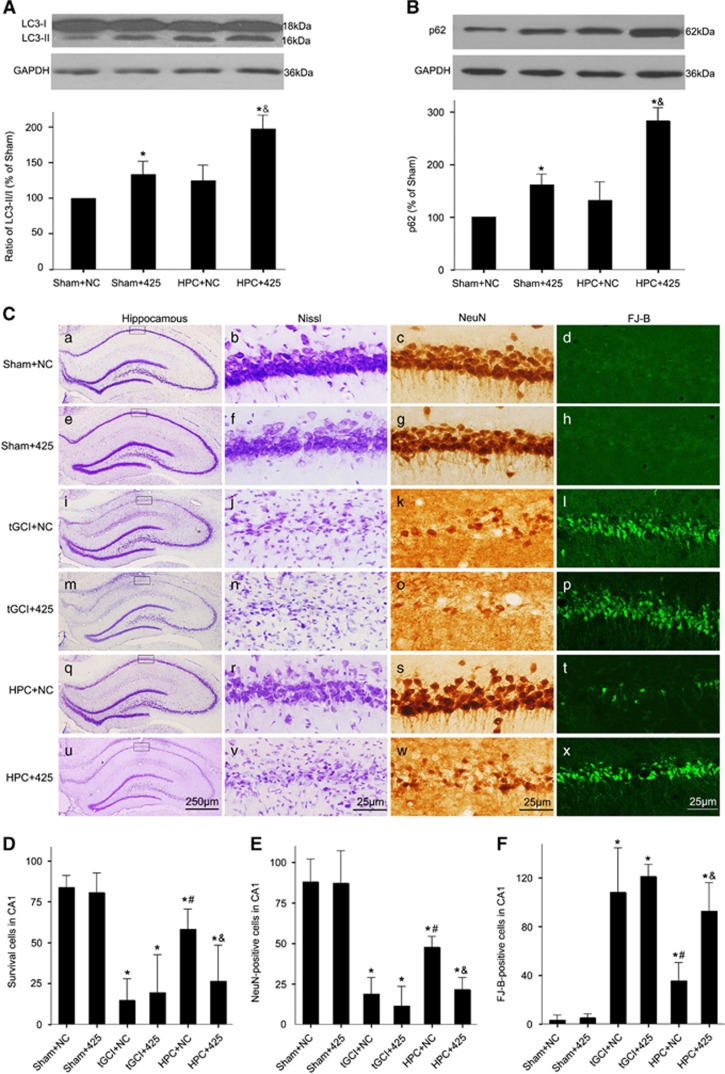
Knockdown of UVRAG decreases autophagic flux and causes neuronal damage in CA1 of hypoxic preconditioned rats. (**A**) The effects of UVRAG knockdown on LC3 in hypoxic preconditioned rats. Same amount of protein lysates from CA1 was subjected to immunoblot analysis using anti-LC3 antibody at 4 h after reperfusion. The histogram presents the quantitative analyses of the ratio of LC3-II/LC3-I. (**B**) The effects of UVRAG knockdown on SQSTM1/p62 in hypoxic preconditioned rats. The histogram presents the quantitative analyses of SQSTM1/p62 levels. Data are expressed as percentage of value of Sham-operated animals. The values are expressed as mean±S.D. *P<0.05 *versus* Sham+NC animals, and ^&^*P*<0.05 *versus* HPC+NC group (*n*=6 in each group). (**C**) Representative microphotographs of cresyl violet staining, immunostaining of NeuN and FJ-B staining showed that knockdown of UVRAG increased neuronal damage in the hippocampus at 7 days after HPC. Sham+NC group (a–d), Sham-operated animals with injection of negative control siRNA vector; Sham+siRNA-425 group, Sham-operated animals with injection of UVRAG-rat-425 vector (e–h); tGCI+NC group (i–l); tGCI+ siRNA-425 group (m–p); HPC+NC group (q–t); HPC+ siRNA-425 group (u–x). Scale bar: a, e, i, m, q, u: 250 *μ*m, b–d, f–h, j–l, n–p, r–t, v–x: 25 *μ*m. (**D**–**F**) Quantitative analyses of survival neurons, NeuN-positive cells and FJ-B-positive cells in CA1. Each bar represents the mean±S.D. *p<0.05 *versus* Sham+NC animals, ^#^*P*<0.05 *versus* tGCI+NC group and ^&^*P*<0.05 *versus* HPC+NC group (*n*=8 in each group)

To test the causal relationship between the UVRAG downregulation and the impairment of AP maturation in CA1 after HPC, we then investigated the impact of UVRAG knockdown on the AP maturation. With UVRAG knockdown both LC3-II/LC3-I ratio and SQSTM1/p62 were remarkably increased either in Sham-operated or HPC rats (Figure 8A and B).

Finally, we examined the effect of UVRAG downregulation on neuroprotection induced by HPC (Figure 8C–F). UVRAG knockdown completely abolished the neuroprotection of HPC, revealed by the percentages of survival cells, NeuN-positive cells and FJ-B-positive cells in CA1. Notably, no obvious cell damage was found in Sham-operated rats either with UVRAG knockdown or the negative control siRNA.

## Discussion

The present study supports the hypothesis that HPC activates Rab7 by mediating UVRAG-Vps16 interaction after tGCI in rats. As a result, the restoration of autophagic flux and promotion of AP maturation, and the ensuing decrease of AP accumulation in CA1 may have contributed to the neuroprotection induced by HPC against tGCI.

HPC has been described as an endogenous strategy by which a sublethal hypoxic exposure can protect brain tissues from further severe ischemic insult.^2, 29, 30^ Several studies have demonstrated that cardioprotection elicited by IPC was mediated via upregulation of autophagy.^31, 32^ An *in vitro* study found that autophagy contributed to IPC-induced neuroprotection against ischemia in PC12 cells.^33^ Also, Sheng *et al.*^34^ reported that the activation of autophagy had an important part in the neuroprotection induced by IPC following focal ischemia in rats. Our previous study demonstrated that the downregulation of autophagy may induce ischemic tolerance after hypoxic postconditioning.^35^ However, the exact effects of autophagy on cerebral ischemia were still controversial.^7, 36, 37, 38, 39, 40, 41^ Undoubtedly, the autophagy pathway is activated after cerebral ischemia.^39, 40, 41, 42^ In this study we have demonstrated that APs and LC3-II increased in CA1 after tGCI. In contrast, HPC decreased APs and LC3-II after tGCI. It is noteworthy that a remarkable increase in ALs was revealed after HPC.

APs can fuse with lysosomes to form ALs where the contents are degraded by lysosomal hydrolases. Therefore, the accumulation of APs after tGCI may imply either an increase in AP formation, or a reduction in autophagic flux. The nucleation and elongation of phagophore are two major steps of autophagy initiation. The latter depends on the transition of LC3-I to LC3-II, which is mediated by Atg5-Atg12 conjugation. Therefore, the loss of Atg5 would effectively inhibit autophagy.^43^ However, there were no significant differences in the level of Atg5 after tGCI with or without HPC. This result indicates that decreased AP accumulation induced by HPC may not be attributed to the suppression of autophagy initiation.

We then examined whether impaired autophagic flux was involved in the accumulation of APs after tGCI. LC3-II is degraded after the fusion of APs with lysosomes. SQSTM1/p62, which is able to bind to ubiquitinated damaged proteins, is designated for autophagic degradation. Therefore, the increase in both the LC3-II/LC3-I ratio and SQSTM1/p62 implies the impairment of autophagic flux.^44^ This study demonstrated increased ratio of LC3-II/LC3-I in CA1 after tGCI, accompanied by an increase of SQSTM1/p62, indicating the autophagic flux was blocked. In contrast, HPC eliminated these effects induced by tGCI. Moreover, both the LC3-II/LC3-I ratio and SQSTM1/p62 remarkably increased after HPC pretreatment with BFA. These results indicate that HPC restored autophagic flux after tGCI.

In addition, the defective AP removal can be caused either by inadequate lysosomal proteolytic activities or defective fusion between APs and lysosomes. Accordingly, levels of the lysosome membrane proteins LAMP2 and the soluble lysosomal enzyme Cathepsin D were examined. The level of LAMP2 did not alter until 48 h of reperfusion after tGCI with or without HPC, suggesting that the size of the lysosomal compartment and total number of lysosomes remained unchanged. Interestingly, LAMP2 was mainly found in neurons at 48 h of reperfusion. However, LAMP2 decreased significantly in neurons at 168 h. Lysosomes are composed of soluble acidic hydrolases, integral membrane proteins and membrane-associated proteins.^45^ Dysfunction in any of these components may cause lysosomal deficits, leading to accumulation of undegraded metabolites. Therefore, decrease of LAMP2 in neurons led to lysosomal dysfunction and ultimately neuronal damage at 168 h after tGCI. Moreover, our results revealed that LAMP2 localized mainly in microglia. Our previous study confirmed that microglia can be activated in CA1 after tGCI.^29^ These findings indicate that the lysosomal activation and alteration in microglia may have played crucial roles in the pathogenesis of tGCI. After tGCI, microglial cells are activated and may attempt to digest damaged components, including debris or misfolded proteins through the autophagy-lysosome pathway. To the best of our knowledge, this is the first report on LAMP2 expression in microglia after cerebral ischemia.

Unlike LAMP2, a persistent increase of Cathepsin D in HPC groups was observed. However, the pretreatment with PA did not block the neuroprotection induced by HPC. These results demonstrate that HPC-induced neuroprotection is neither mediated via the increase in the number of lysosome nor the improvement of lysosomal function after tGCI. Taken together, a defect in autophagosome-lysosome fusion may have caused AP accumulation after tGCI. Indeed, as compared with HPC rats, the number of total autophagic vacuoles in CA1 of tGCI rats substantially increased while the number of ALs did not, suggesting impaired autophagosome-lysosome fusion after tGCI. Furthermore, the inhibition of autophagosome-lysosome fusion via CQ remarkably aggravated neuronal death in CA1, with increases in both the ratio of LC3-II/LC3-I and SQSTM1/p62. These data indicates that AP maturation may have contributed to the HPC-induced neuroprotection against neuronal injury with tGCI.

The regulation of AP maturation induced by HPC is still poorly understood. In an *in vitro* study, Rab7 was shown to be involved in the regulation of autophagosome-lysosome fusion and lysosomal genesis.^5^ The upregulation of Rab7 has an important role in mediating autophagic flux increases induced by starvation in cardiomyocytes.^46^ Besides, the downregulation of Rab7 impairs AP maturation and exacerbates cell death in cultured cardiomyocytes.^47^ These studies support the involvement of Rab7 in the regulation of AP maturation. However, our results failed to demonstrate any effect of HPC on the expression of Rab7 during reperfusion. Noteworthily, the distribution of Rab7 between ischemic and HPC brains was different after reperfusion. Rab7 of CA1 in tGCI rats was expressed mainly in glia at 168 h of reperfusion. Alternatively, most Rab7 in HPC was expressed in neurons. This is the first report on the transition of Rab7 expression from neurons to astrocytes after tGCI. It remains unclear whether this difference of Rab7 distribution between HPC and tGCI rats contributes to neuroprotection and AP maturation induced by HPC.

Rab7 activity depends on its functional states. In an *in vitro* study with cultured Purkinje neurons, Rab7 activity decreased after prolonged trophic factor withdrawal, with AP accumulation and cell death. However, the deactivation of Rab7 was prevented and autophagic flux was restored by adding insulin-like growth factor-I.^48^ Our study showed that the RILP-Rab7 interaction in HPC rats was higher than that in tGCI, revealing that HPC enhanced Rab7 activity during tGCI-induced autophagy activation.

The C-Vps complex is thought to be a GEF of Rab7 and promotes Rab7 transiting from GDP-bound to GTP-bound state, resulting in activation of Rab7.^49^ Moreover, the Vps39p subunit of HOPS complex has GEF activity on Ypt7p, the yeast ortholog of Rab7.^24^ In this study, the interaction of Vps16 with Rab7 in tGCI rats was enhanced after HPC. This interaction may have mediated the activation of Rab7 after HPC. Previous evidence indicated that the C-Vps complex was positively regulated by UVRAG^25^ and UVRAG had a vital role in autophagosome-lysosome fusion.^25, 50^ Therefore, it is speculated that UVRAG interaction with the C-Vps complex may enhance the ability of C-Vps in GDP-to-GTP conversion of Rab7, thereby promoting the fusion of APs with lysosomes. Our results support that UVRAG deficiency disrupts the interaction between Vps16 and Rab7, decreases the activation of Rab7, prevents AP fusion with lysosomes and ultimately abrogates the neuroprotection provided by HPC.

In summary, we found that HPC attenuated neuronal death by enhancing AP maturation in CA1 after tGCI. The activation of Rab7 played an essential role in AP maturation, which mediated neuroprotection of HPC against tGCI. Moreover, given that UVRAG deficiency disrupted the interaction between Vps16 and Rab7 and decreased the activation of Rab7, UVRAG might be deemed as a potential molecular target for protection against tGCI.

## Materials and methods

All surgical procedures and animal experiments were performed according to the ARRIVE guidelines, and were approved and monitored by the Animals Care and Use Committee of Guangzhou Medical University. Adult male Wistar rats weighing 220–280 g (7–8 weeks old) were obtained from Southern Medical University. Rats for experiment were housed under standard temperature (22±1 °C) in a 12 h light/dark controlled environment with free access to food and water.

In this study, 442 rats were used, and nine of which in tGCI and five in HPC groups died during the procedure of tGCI, and three in tGCI and two in HPC groups died after tGCI. Also, three rats died after intracerebroventricular injection of BFA; five died after the injection of CQ; six died after the injection of HVJ-E vectors, and two died during the procedure of hypoxia. Nine rats that convulsed during ischemia and four during 72 h post-ischemia were also excluded.

### Transient global ischemia and HPC

To induce acute cerebral ischemia, tGCI was carried out according to the four-vessel occlusion method.^51^ All procedures in our study were performed under aseptic conditions. In brief, anesthesia was induced with 3–4% isoflurane in a chamber and maintained during the operation with a mask using 2–3% isoflurane. Vertebral arteries were electrocauterized, and common carotid arteries were isolated. A teflon/silastic occluding device was placed loosely around each carotid artery without interrupting carotid blood flow. Forebrain ischemia was induced 24 h after surgery in the rats which were awake, by occluding both common carotid arteries for 10 min. After occlusion, rats that lost their righting reflex within 1 min and whose pupils dilated were selected for experiments. Rectal temperature was maintained at 37~38 °C throughout the procedure. Sham-operated rats received the same surgical procedure except the occlusion of arteries. Rats that convulsed during ischemia or post-ischemia were excluded from this study.

Twenty-four hours before ischemia, rats were placed in a hypoxia chamber through which air containing 8% O_2_ and 92% N_2_ flowed continuously at the temperature of 23–25 °C, and preconditioned for 30 min.^2^

### Pharmacological interventions

The implantation of the intracerebroventricular injection cannula into the right lateral ventricle was performed stereotaxically under isoflurane anesthesia. The cannula was stereotaxically placed through a burr hole opened on the right parietal skull at 1.5 mm lateral, 0.8 mm posterior and 4.0 mm dorsal with respect to the bregma, and affixed to the skull with stainless steel screws and cranioplastic cement. Rats were allowed to recover from surgery for one week before treatment. All animals displayed normal feeding and drinking behaviors postoperatively. For pharmacological interventions, BFA (4 *μ*mol/l, 10 *μ*l, intracerebroventricularly, Sigma Aldrich, St Louis, MO, USA.) was administered at 30 min before tGCI or HPC and PA (32 *μ*g/kg, intraperitoneally, Sigma), CQ (60 mg/kg, intraperitoneally, Sigma) or the vehicle (25% dimethylsulfoxide in phosphate-buffered saline (PBS)) was administered at 2 h before HPC. To evaluate the toxicity of PA and CQ to the cells of hippocampus, six animals without hypoxia or ischemia were treated with PA and CQ intraperitoneally, respectively.

### Assessment of cellular damage

As studied previously,^2^ Nissl, FJ-B and NeuN staining were performed to determine the hippocampal cell damage at 7 days after reperfusion. The sections from Nissl and NeuN staining were examined under a light microscope (× 660). FJ-B stained images were observed with a fluorescence microscope (Leica Microsystems, Wetzlar, Hessen, Germany). Cell counts were conducted as described previously.^52^ Cells in the CA1 pyramidal layer were quantitatively analyzed within three non-repeated rectangular areas of 0.037 mm^2^ in the typical dorsal hippocampus between stereotaxic planes AP 4.5 and 4.9 mm (interaural). Data were quantified bilaterally in sections from each brain and assessed double-blindedly. Also, four sections per animal were evaluated.

### TEM

For electronic microscopic analysis, samples were pre-fixed in 0.1 M sodium cacodylate-buffered (PH 7.4) 2.5% glutaraldehyde solution for 2 h and post-fixed in 0.1 M sodium cacodylate-buffered 1% O_s_O_4_ solution for 1 h. After dehydrated in a step-wised ethanol gradient solution (50% ethanol 20 min, 70% ethanol 20 min, 80% ethanol 20 min, 90% ethanol 20 min, 95% ethanol 20 min and 100% ethanol 20 min), samples were incubated with propylene oxide for 10 min, then impregnated with a mixture of propylene oxide/Spurr (1:1) and embedded in Spurr resin. Ultrathin sections were examined using JEOL JEM-1200EX TEM. Five rats were used at each experimental group. Four tissue samples were taken for thin sections from each animal. Five sections were cut per sample. Ten electron micrographs taken from the cell layer areas between the nuclei and apical dendrites of the CA1 pyramidal neurons were taken per section at a primary magnification of × 25000. Morphometrical measurements of APs and ALs were carried out using the point-counting method.^44, 53^ Data from each electron micrograph taken from the samples of the same experimental point were used to calculate the mean and its variation. The cytoplasmic volume fractions of the autophagic vacuole were expressed as percentage of the cytoplasmic volume.

### Immunohistochemistry

The animals were killed at 0, 4, 24, 48 and 168 h after reperfusion with or without hypoxia (*n*=6 in each group). Single-label immunohistochemistry was conducted by the avidin-biotin-peroxidase complex method.^2^ The primary antibodies used in these studies include LC3 (1:100; NOVUS, Littleton, CO, USA), Atg5 (1:100; Abcam, Cambridge, UK), LAMP2 (1:2000; Sigma), Cathepsin D (1:1000; Sigma), Rab7 (1:100; Abcam), UVRAG (1:100; Millipore, Bedford, MA, USA) and NeuN (1:4000; Millipore). Immunopositive cells in which the reaction product was present within a clear and regular-shaped cytoplasmic or nuclear border were quantified under a light microscope with × 660 magnification. The average intensity of Cathepsin D-positive fiber staining in which reaction product presented in the cell processes in the CA1 subregion was determined using Image-Pro Plus software for Windows, version 6.0 (Media Cybernetics, Inc. Warrendale, PA, USA). Four non-repeated × 200 magnification microscopic random fields (141.15 *μ*m^2^ per field) in the pyramidal layer, stratum radiatum and stratum lacunosum-moleculare of each subject were assessed in four coronal tissue sections. Measures of staining intensity of fibers were averaged across tissue sections to provide a single mean value for each structure for each rat. These mean values were used for statistical analysis.

Double-fluorescent immunohistochemistry was conducted to demonstrate cell types and the exact position where LAMP2 and Rab7 were expressed. NeuN, MAP-2, GFAP, OX-42 and Iba-1 were used to identify NeuN, neuronal cell bodies, astrocyte and microglia, respectively. Double-fluorescent immunohistochemistry was performed as described previously.^2^ Antibodies used in these studies include LAMP2 (1:1000; Sigma), Rab7 (1:50; Abcam), NeuN (1:1000; Millipore), GFAP (1:2000; Millipore), OX-42 (1:100; Millipore), Iba-1 (1:100; Millipore), MAP-2 (1:300; Millipore), Cy3-conjugated goat anti-mouse IgG antibody (1:100; Invitrogen, Carlsbad, CA, USA) and FITC-conjugated goat anti-rabbit antibody (1:50; Invitrogen). Slides were analyzed with a confocal laser microscope (Leica Microsystems).

### Western blotting

Rats were killed under chloral hydrate anesthesia at 0, 4, 24 and 48 h after reperfusion with or without hypoxia (*n*=5 in each group). The brain tissue was cut into 2 mm coronal slices using a brain matrix and the CA1 subregions of bilateral hippocampi were quickly dissected under a stereomicroscope. Proteins of the hippocampal CA1 subregion were extracted as described previously.^2^ Western blot was performed as described previously^2^ using the antibodies including LC3 (1:10000; Novus), Atg5 (1:1000; Abcam), SQSTM1/p62 (1:2000; Abcam), LAMP2 (1:6000; Sigma), Cathepsin D (1:1000; Sigma), Rab7 (1:1000; Abcam), Vps16 (1:1000; Proteintech Group, Inc. Chicago, IL, USA), UVRAG (1:1000; Millipore) and GAPDH (1:10000; Proteintech). Densitometric analysis for the quantification of the bands was performed using image analysis software (Quantity One, Bio-Rad Laboratories, Inc., Hercules, CA, USA). Relative optical densities of protein bands were calibrated with *β*-actin or GAPDH and normalized to those in Sham-operated rats.

### Immunoprecipitation

Tissues from the CA1 subregions of bilateral hippocampi were lysed in a buffer composed of 50 mM Tris-HCl (pH 7.5), 150 mM NaCl, 0.5% Triton X-100 and 1 mM EDTA supplemented with a protease inhibitor tablet (Roche, Mannheim, Germany). A total of 0.4 mg of protein was incubated with 8 *μ*g of RILP antibody (ProSci, San Diego, CA, USA) or Vps16 antibody (Proteintech) overnight at 4  °C followed by incubation with 120 *μ*l of protein G-Sepharose (Millipore) for 2 h at 4 °C. Immunocomplexes were collected by centrifugation and examined with western blot after separation by SDS-PAGE.

### Reverse transcription quantitative RT-PCR

Total RNA was extracted from RGC-5 transfected with siRNAs using Trizol reagent (Fermentas, Pittsburgh, PA, USA). RT-qPCR was performed according to the standard protocol. The primers of UVRAG were 5′-ACTCCAGACTTGAGGCAAAC-3′ (forward) and 5′-ACAGATACTCACCATCTGACC-3′ (reverse). The primers of *β*-actin were 5′-AGGGAAATCGTGCGTGACAT-3′ (forward) and *β*-actin-R 5′-GAACCGCTCATTGCCGATAG-3′ (reverse). Quantitative PCRs were conducted by LightCycler Fast-Start DNA Master SYBR Green 1 kit and on a LightCycler 1.5 PCR machine (Roche). Data were analyzed using the comparative *C*_*t*_method (2^-ΔΔ*Ct*^) and normalized to *β*-actin. All reactions were performed in triplicate. Results were expressed as fold changes compared to negative control.

### UVRAG RNAi knockdown

Three different sequences of UVRAG siRNAs (UVRAG-rat-558: 5′-GCUGUUGAUAGAGUGGAAATT-3′, 5′-UUUCCACUCUAUCAACAGCTT-3′

UVRAG-rat-371: 5′-CCUACUUCACUCUUCAUUUTT-3′, 5′-AAAUGAAGAGUGAAGUAGGTT-3′

UVRAG-rat-425: 5′-GGAGUGAAGUGAUUAAGAATT-3′, 5′-UUCUUAAUCACUUCACUCCTT-3′) and negative control siRNA (5′-UUCUCCGAACGUGUCACGUTT-3′, 5′-ACGUGACACGUUCGGAGAATT-3′) were purchased from ViewSolid Biotech (Beijing, China). To select the highest knockdown effects of UVRAG siRNAs, three UVRAG siRNA sequences at 25 nM, 50 nM and 100 nM were transferred into rat RGC-5. Then, UVRAG mRNA expression levels were detected by RT-qPCR in transferred cell lysates, and the UVRAG siRNAs with the lower express levels of UVRAG mRNA were selected for transferring into rat brain. Hemagglutinating virus of Japan HVJ-E vector kit (CosmoBio, Tokyo, Japan) was used to deliver of siRNA plasma into target tissues. Following with manufacturer’ protocols, HVJ-E was incorporated with UVRAG siRNAs to yield an HVJ-E UVRAG siRNA complex (vector). The vectors were introduced into target tissues by membrane-fusion activity of fusion protein. Four *μ*g of UVRAG siRNAs or negative control siRNA were combined with HVJ-E, respectively. The complexes in PBS were administered bilaterally into the hippocampal CA1 region of rats by stereotaxic injection 60 min before HPC. A 5-*μ*l volume containing 2 *μ*g of siRNA was injected into the CA1 region of dorsal hippocampus (3.5 mm posterior to bregma, 2.3 mm lateral to bregma, 2.6 mm below the dura) using a 10-*μ*l Hamilton syringe with 34-gauge needle at a flow rate of 0.3 *μ*l/min. For immunoblot analysis, rats were killed at 4 h after reperfusion in HPC or 24 h after injection of vectors in Sham-operated group.

### Data analyses

Data were expressed as mean±S.D. Statistical significance was determined by one-way ANOVA or the two-tailed Student’s *t*-test. The Mann–Whitney *U-*test was used for morphometrical measurements of TEM. A two-tailed *P*-value<0.05 was considered statistically significant.
