# Supplementary material for: Autophagosome maturation mediated by Rab7 contributes to neuroprotection of hypoxic preconditioning against global cerebral ischemia in rats
Source: Cell Death Dis. 2017 Jul 20;8(7):e2949–. doi: 10.1038/cddis.2017.330 (PMC5550874; doi:10.1038/cddis.2017.330)
Supplement: Supplementary Figure 1 Legend [file cddis2017330x1.docx]

**Figure Legends for Supplementary Material**

**Fig**I**.** The effect of hypoxic preconditioning on expression of Atg5 in CA1 after tGCI. **(A)** Immunohistochemistry for Atg5 in the hippocampus after tGCI with or without HPC. Representative images show Sham-operated group (a and b), 48 hours after reperfusion of tGCI group (c and d), 48 hours after reperfusion of HPC group (e and f), 7 days after reperfusion of tGCI group (g and h), and 7 days after reperfusion of HPC group (i and j), respectively. Scale bar: a,c,e,g,i: 250 μm; b,d,f,h,j: 25 μm.**(B)** Quantitative analysis of immunoreactive cell counting of Atg5 in CA1. Data are shown as mean±S.D. (n=6 in each group). **(C)** Western blot analysis of Atg5 in CA1 of ischemic and hypoxic preconditioned rats. The histogram presents the quantitative analyses of Atg5 levels. Data are expressed as percentage of value of Sham-operated animals. Each bar represents the mean±S.D. (n=5 in each group).
