# Supplementary figures and images for: Autophagosome maturation mediated by Rab7 contributes to neuroprotection of hypoxic preconditioning against global cerebral ischemia in rats
Source: Cell Death Dis. 2017 Jul 20;8(7):e2949–. doi: 10.1038/cddis.2017.330 (PMC5550874; doi:10.1038/cddis.2017.330)

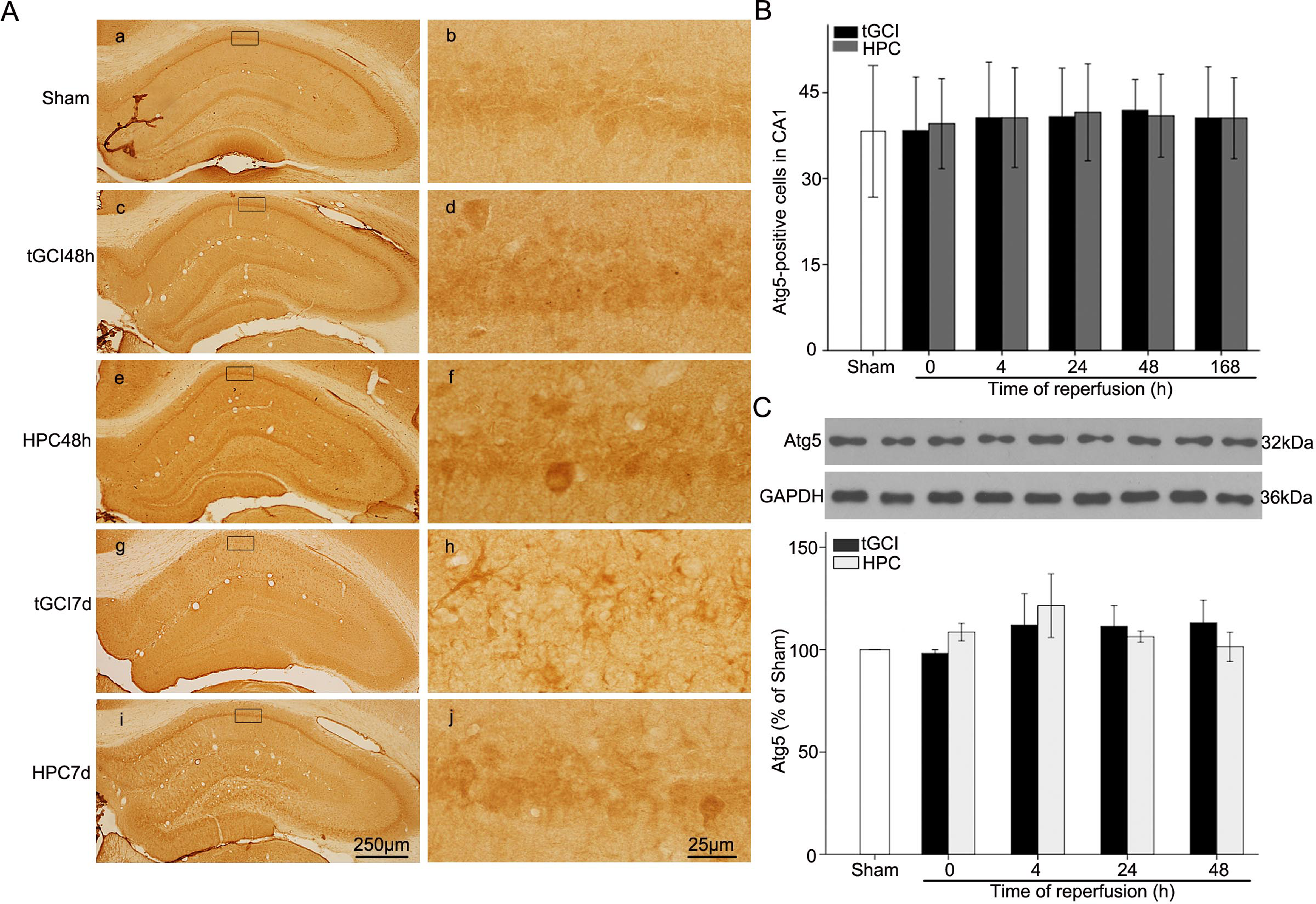

Supplement: Supplementary Figure 1 [file cddis2017330x2.tif]
